# Supplementary material for: Central role of Prominin-1 in lipid rafts during liver regeneration
Source: Nat Commun. 2022 Oct 20;13:6219. doi: 10.1038/s41467-022-33969-4 (PMC9585078; doi:10.1038/s41467-022-33969-4)
Supplement: Supplementary file 1 — Supplementary information [file 41467_2022_33969_MOESM1_ESM.pdf]

Supplementary information

Central role of Prominin-1 in lipid rafts during liver regeneration

Myeong-Suk Bahn[1*], Dong-Min Yu[1*], Myoungwoo Lee[1], Sung-Je Jo[1], Ji-Won Lee[1], Ho-Chul Kim[1], Hyun Lee[1], Hong Lim Kim[2], Arum Kim[1], Jeong-Ho Hong[1], Jun Seok Kim[1], Seung-Hoi Koo[1], Jae-Seon Lee[3], and Young-Gyu Ko[1¶]

[1]Division of Life Sciences, Korea University, Seoul, 02841, Korea, [2]Laboratory of Electron Microscope, Integrative Research Support Center, College of Medicine, The Catholic University of Korea, Seoul, Korea, [3]Research Center for Controlling Intercellular Communication, College of Medicine, Inha University, Incheon, 22212, Korea

*These authors contributed equally.

[¶]To whom correspondence should be addressed

Young-Gyu Ko, Ph. D.

Division of Life Sciences, Korea University

145, Anam-ro, Seongbuk-gu, Seoul, 02841, Korea

e-mail: ygko@korea.ac.kr; TEL: 82-2-3290-3453

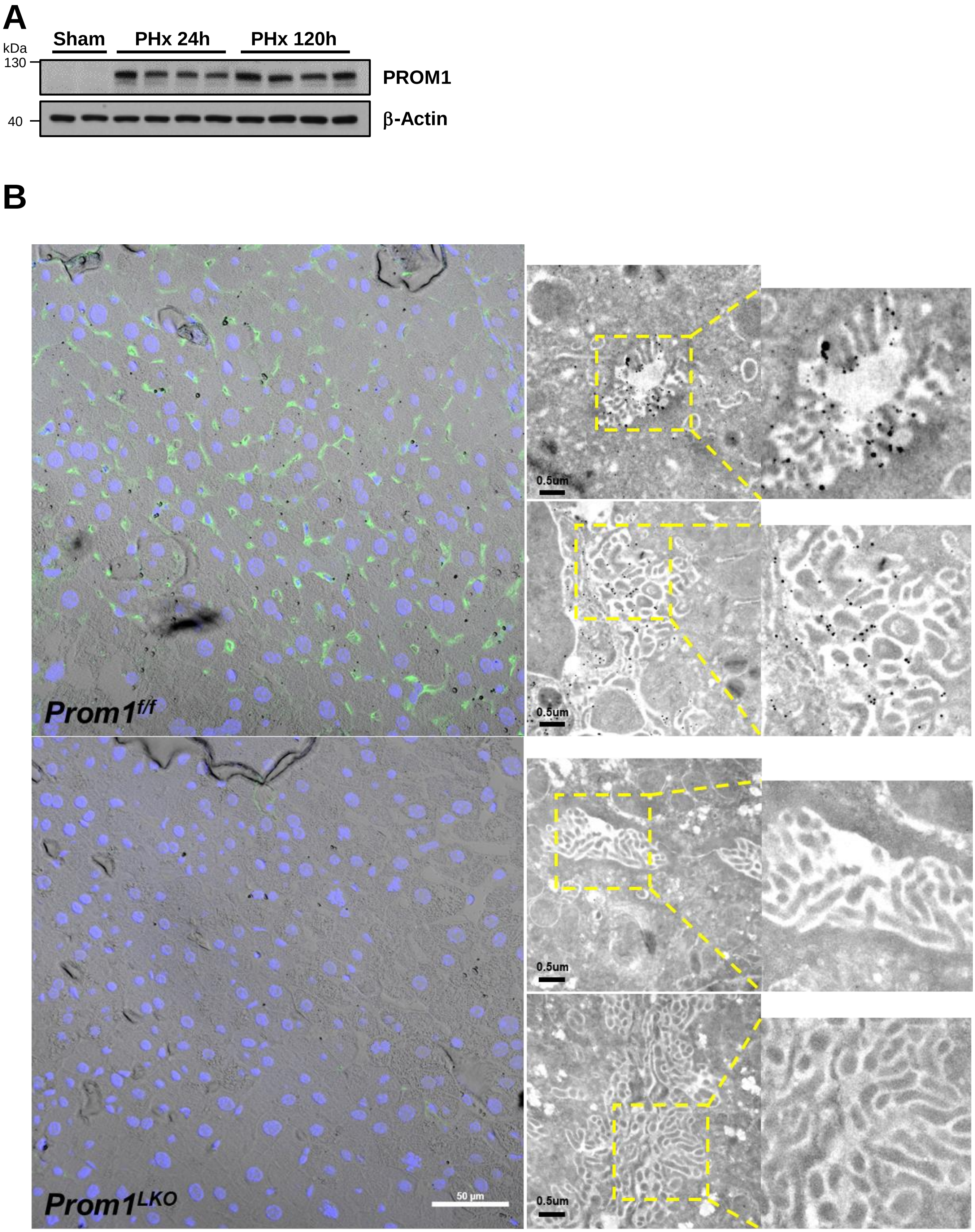
A
Sham
PHx 24h
PHx 120h
kDa
130
40
PROM1
β-Actin
B
Prom1f/f
Prom1LKO
50 μm
0.5um
0.5um
0.5um
0.5um

Supplementary Figure 1. PROM1 protein level is increased in liver after partial hepatectomy. (A) Immunoblotting for PROM1 in wild type livers 24 hours and 120 hours after PHx (N=2 for sham, N=4 for PHx 24h and 120h). **(B)** Correlative light electron microscopy (CLEM) showed PROM1 expression in the microvilli of hepatocytes in PHx livers. PROM1 was labeled with a monoclonal rat antibody (13A4) and was visualized using an Alexa Fluor 488-Fluoro Nanogold (Nanoprobes). The left panels showed the immunofluorescence signals of PROM1 in PHx liver sections using a confocal microscopy (Scale bar = 50um). The right panels showed the subcellular localization of immuno-gold labeled PROM1 in the sections (Scale bar = 0.5um). The yellow dotted boxes were enlarged. Source data are provided as a Source Data file.

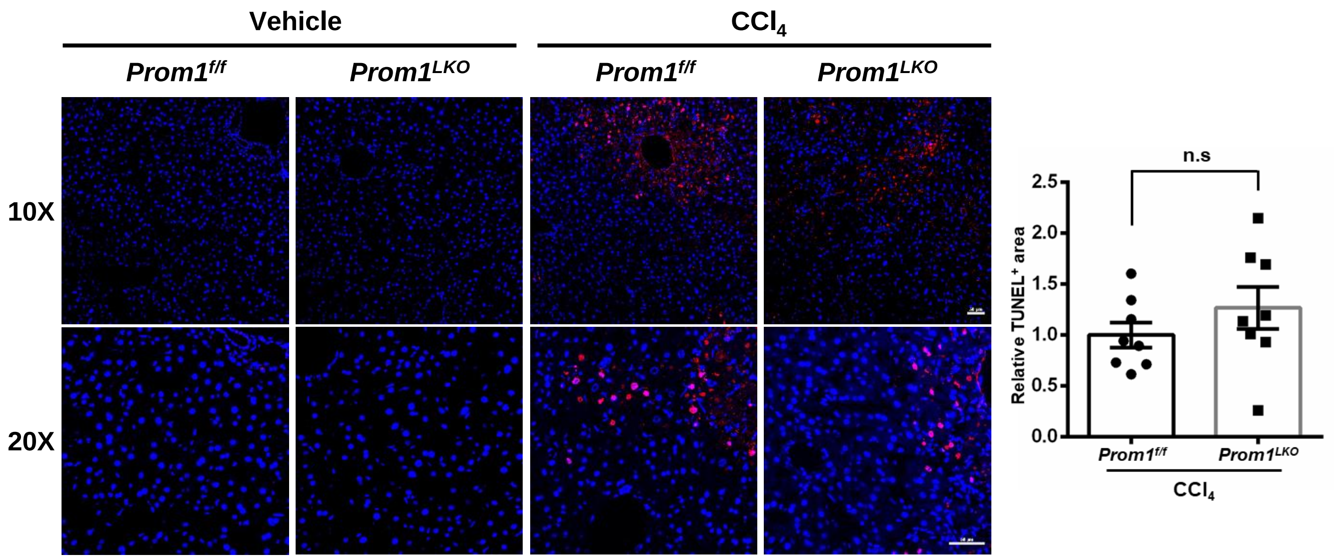

Supplementary Figure 2. PROM1 deficiency does not change CCl_4-induced apoptosis.

CCl_4-induced apoptosis was analyzed in 8-week-old male *Prom1$^{f/f}$* and *Prom1^{LKO}* mice livers by TUNEL assay after CCl_4 injection for 48 hours. The relative TUNEL-positive area was analyzed statistically in 8 images per group (n=3 per each group). The relative TUNEL-positive area was normalized to the number of DAPI-stained dots. Scale bar = 50 µm. Two-sided student *t*-test; n.s, nonsignificant. Data are expressed as the mean ± SEM with individual values. Source data are provided as a Source Data file. TUNEL, terminal deoxynucleotidyl transferase dUTP nick end labeling

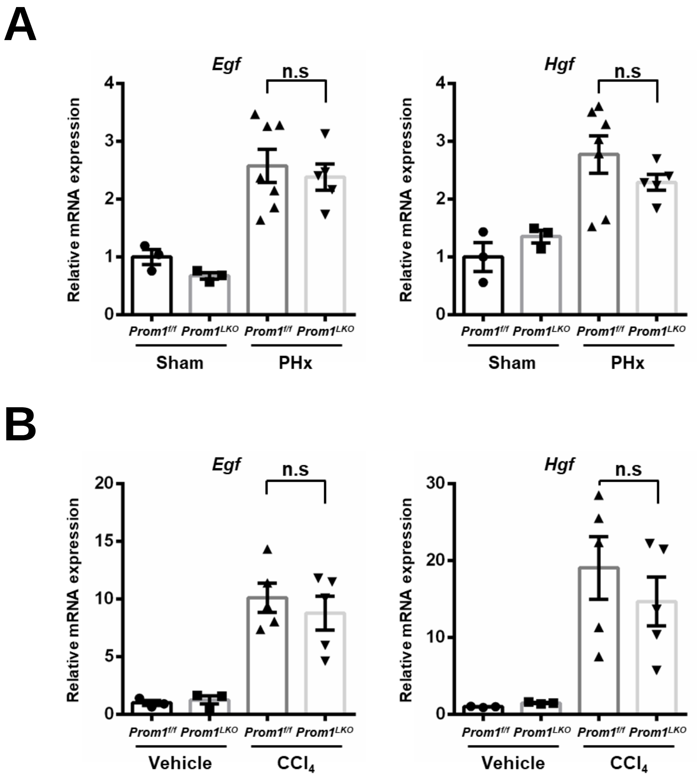

Supplementary Figure 3. PROM1 deficiency does not change the expression of *Egf* and *Hgf* after both PHx and CCl_4 injection. The relative mRNA levels of *Egf* and *Hgf* in the liver 24 hours after PHx **(A)** and 48 hours after CCl_4 injection **(B)**. Each mRNA level was normalized by 18S rRNA. n=3 for sham and vehicle, n=7 for $Prom1^{f/f}$ PHx, n=5 for $Prom1^{LKO}$ PHx, $Prom1^{f/f}$ CCl_4 and $Prom1^{LKO}$ CCl_4. Two-sided student *t*-test; n.s, nonsignificant. Data are expressed as the mean ± SEM with individual values. Source data are provided as a Source Data file.

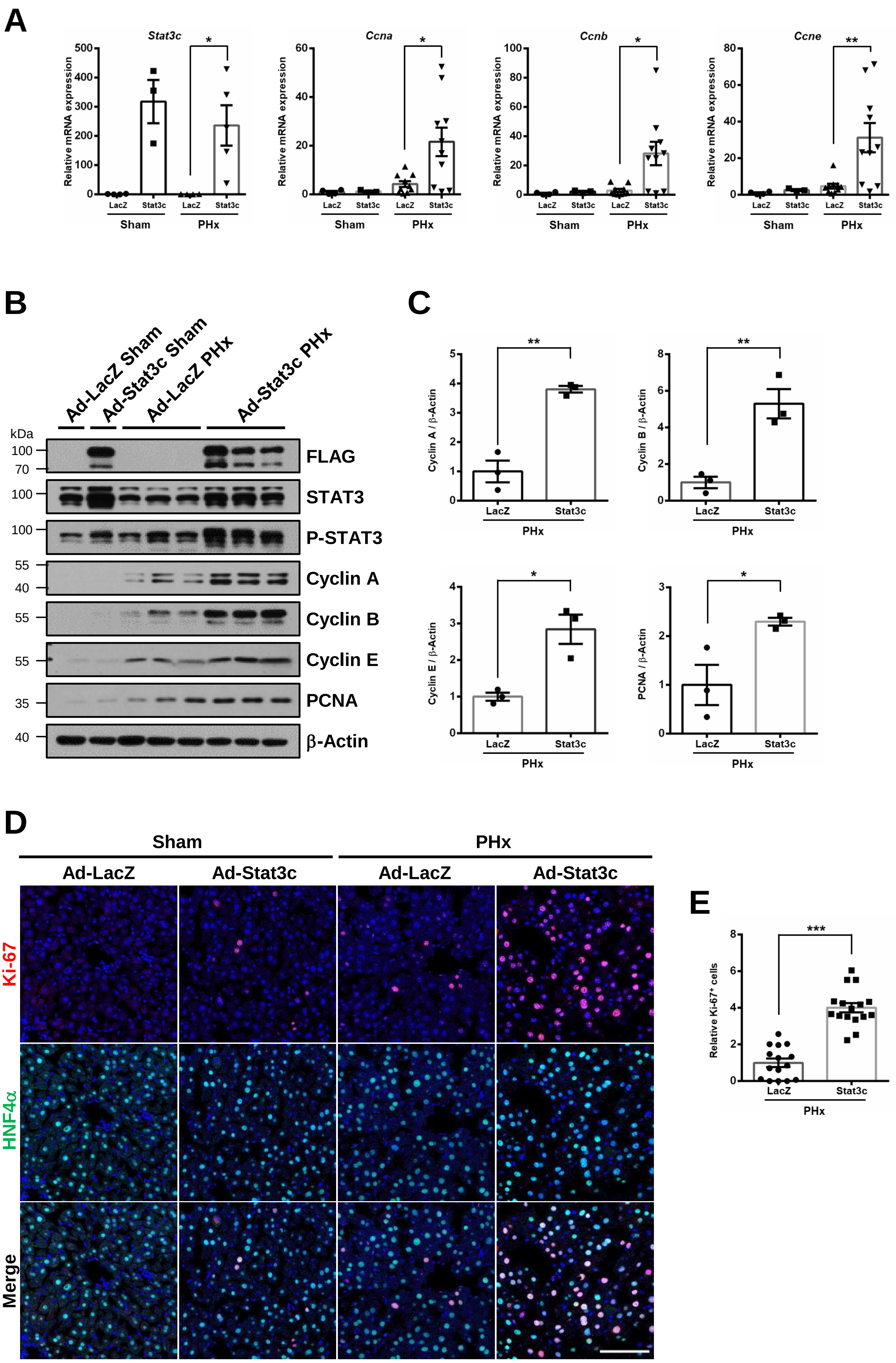
A
Stat3c
Ccna
Ccnb
Ccne
Relative mRNA expression
LacZ
Stat3c
Sham
PHx
*
**
B
Ad-LacZ Sham
Ad-Stat3c Sham
Ad-LacZ PHx
Ad-Stat3c PHx
kDa
FLAG
STAT3
P-STAT3
Cyclin A
Cyclin B
Cyclin E
PCNA
β-Actin
C
Cyclin A / β-Actin
Cyclin B / β-Actin
Cyclin E / β-Actin
PCNA / β-Actin
D
Sham
PHx
Ad-LacZ
Ad-Stat3c
Ki-67
HNF4α
Merge
E
Relative Ki-67⁺ cells
***

Supplementary Figure 4. The expression of STAT3C rescues liver regeneration in liver-specific *Prom1*-deficient mice. A 2/3 partial hepatectomy was performed in 8-week-old male *Prom1*^{LKO} mice after infection with adeno-LacZ or adeno-Stat3c-FLAG. **(A)** The relative mRNA levels of *Stat3c*, *Ccne*, *Ccna*, and *Ccnb* in the liver 24 hours after PHx. Each mRNA level was normalized by 18S rRNA. n=3 for sham, n=10 for PHx. p=0.016 for *Ccna*, p=0.011 for *Ccnb*, p=0.009 for *Ccne*. **(B, C)** Immunoblotting for cyclin A, B and E, PCNA, FLAG, STAT3 and P-STAT3 in the liver 48 hours after PHx (B). Statistical analysis of the band intensities of cyclins A, B and E and PCNA in B. The band intensity of each protein was normalized to that of β-actin. n=3 independent samples, p=0.002 for Cyclin A, p=0.007 for Cyclin B, p=0.011 for Cyclin E, p=0.037 for PCNA (C). **(D)** Double immunofluorescence for Ki-67 and HNF4α in the liver 48 hours after PHx. (E) Statistical analysis of the number of Ki-67-expressing cells (n=3 independent mice, 5 fields per mouse, $p=1.297\times10^{-9}$). The number of Ki-67-positive cells was normalized to the number of DAPI-stained dots. Scale bar = 100 µm. Two-sided student *t*-test; $*p < 0.05$, $**p < 0.01$, $***p < 0.001$. Data are expressed as the mean ± SEM with individual values. Source data are provided as a Source Data file.

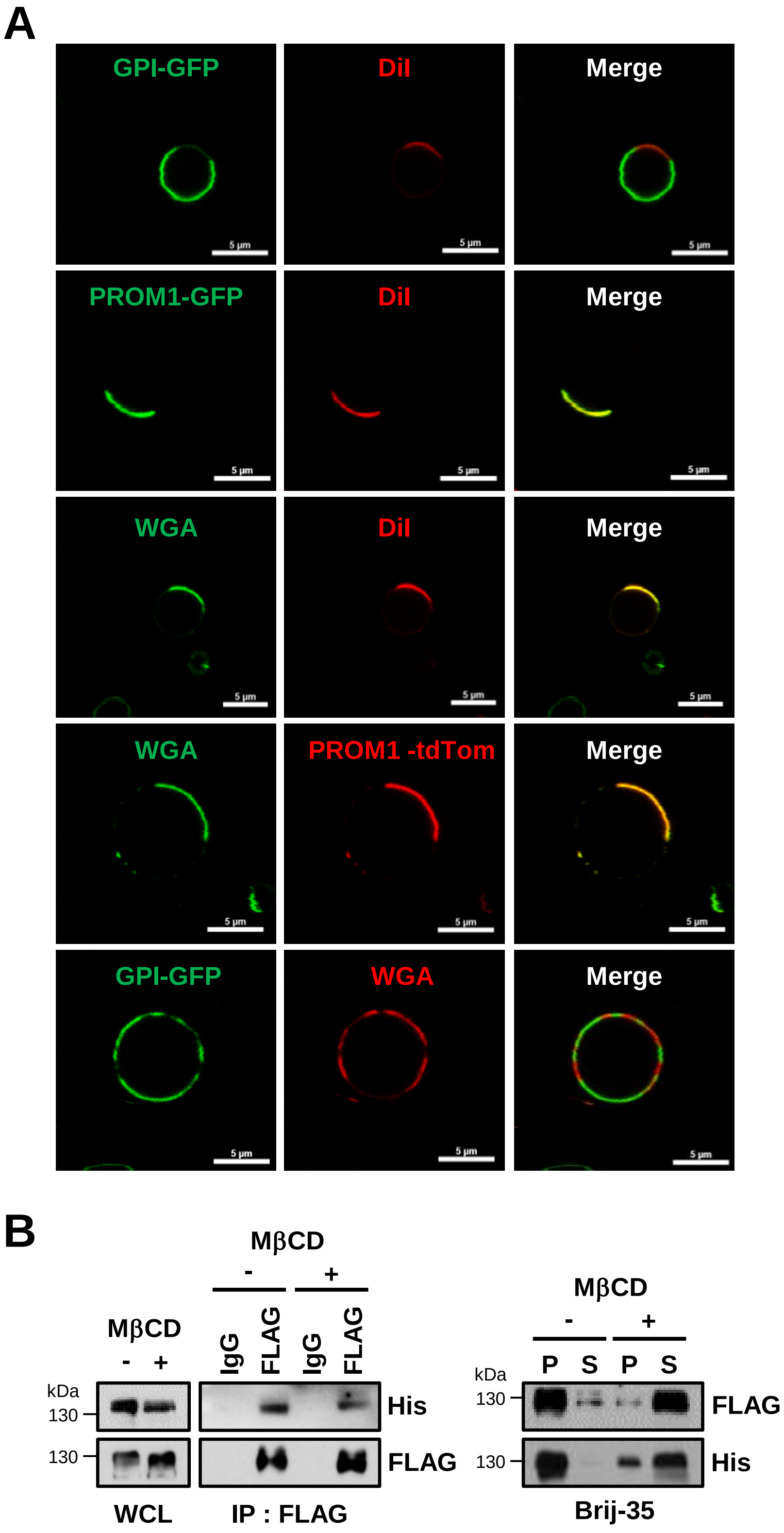
A
GPI-GFP
DiI
Merge
PROM1-GFP
DiI
Merge
WGA
DiI
Merge
WGA
PROM1 -tdTom
Merge
GPI-GFP
WGA
Merge
5 μm
B
MβCD
- +
kDa
130
130
WCL
MβCD
- +
IgG FLAG IgG FLAG
His
FLAG
IP : FLAG
MβCD
- +
P S P S
kDa
130
130
FLAG
His
Brij-35

Supplementary Figure 5. The molecular interaction between PROM1 and GP130 is not limited in lipid rafts. (A) GPMVs were isolated from HEK 293 cells expressing GPI-GFP, PROM1-GFP or -tdTom. DiI (as a non-raft marker) and WGA (as a glycoprotein marker) staining was performed before GPMV isolation. Scale bar = 5um. **(B)** The molecular interaction between PROM1 and GP130 was determined by co-immunoprecipitation from HEK 293 cells overexpressing PROM1-FLAG and GP130-His after 10mM MβCD treatment for 1 hour. The membrane cholesterol depletion was confirmed by immunoblotting for PROM1-FLAG and GP130-His from Brij-35 soluble (supernatant, S) and insoluble (pellet, P) fractions after 17,000g 10min centrifugation (Right panel). Co-immunoprecipitation was performed from NP-40 lysates (Left panel). WCL, whole cell lysates; IP, immunoprecipitation; IgG, normal IgG. Source data are provided as a Source Data file.

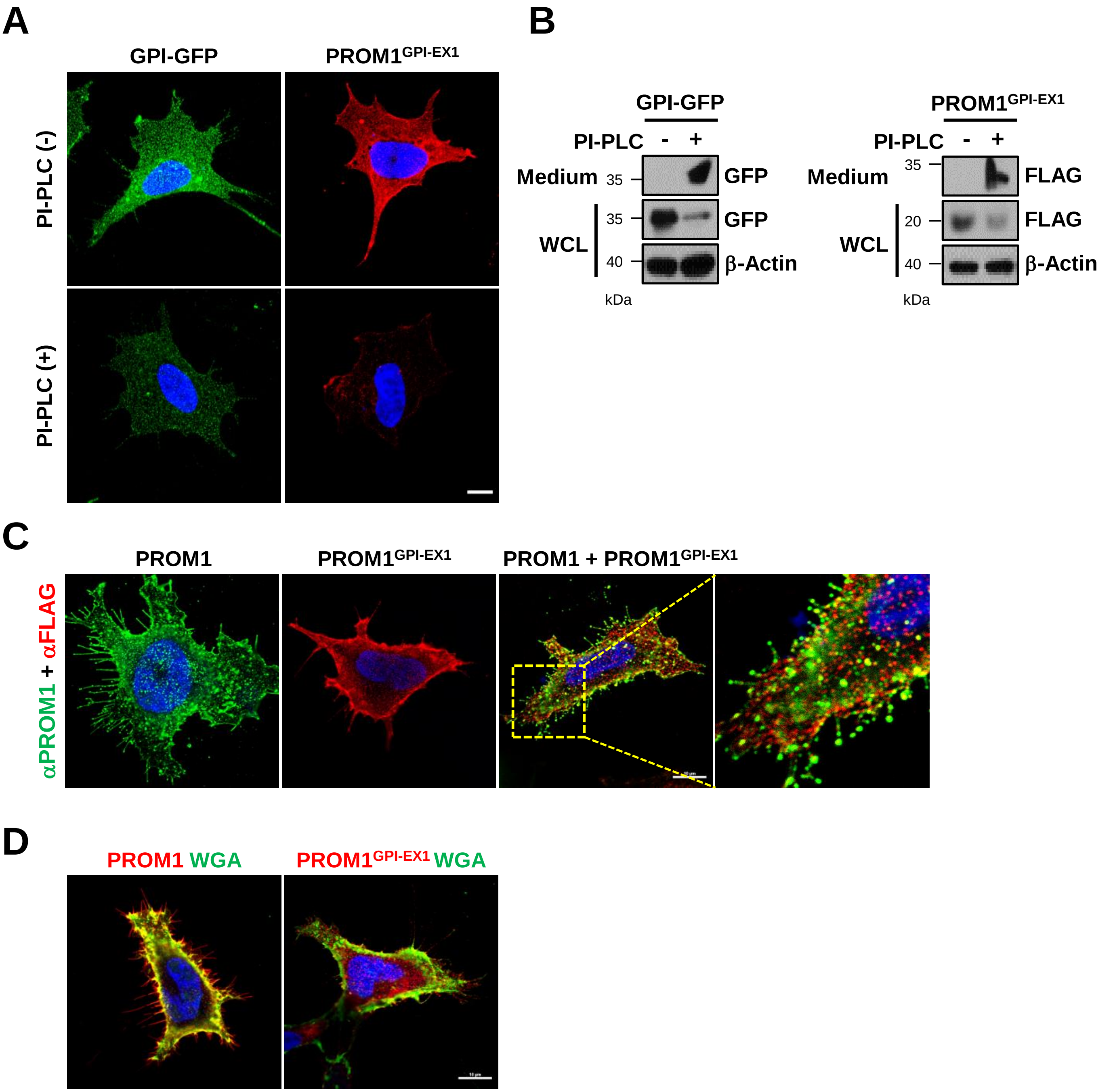

Supplementary Figure 6. PROM1 and PROM1$^{GPI\text{-}EX1}$ is not co-localized in the same type of lipid rafts. (A, B) HEK 293 cells were transfected with GPI-GFP or FLAG-tagged PROM1$^{GPI\text{-}EX1}$ vector. After 24 hours, PI-PLC (1U/ml) was treated for 1 hour at 37°C. GPI-GFP was used as a positive control. The surface immunofluorescence for GFP or FLAG after PI-PLC treatment (A). GFP, FLAG, and b-actin in whole cell lysates (WCL) and media after PI-PLC treatment were determined by immunoblotting (B). Scale bar = 10um. **(C, D)** HEK 293 cells were transfected with untagged PROM1 and/or FLAG-tagged PROM1$^{GPI\text{-}EX1}$ for 24 hours. Double surface immunofluorescence for PROM1 and FLAG (C). The surface immunofluorescence for PROM1 or FLAG after WGA staining (D). Scale bar=10um. Source data are provided as a Source Data file.

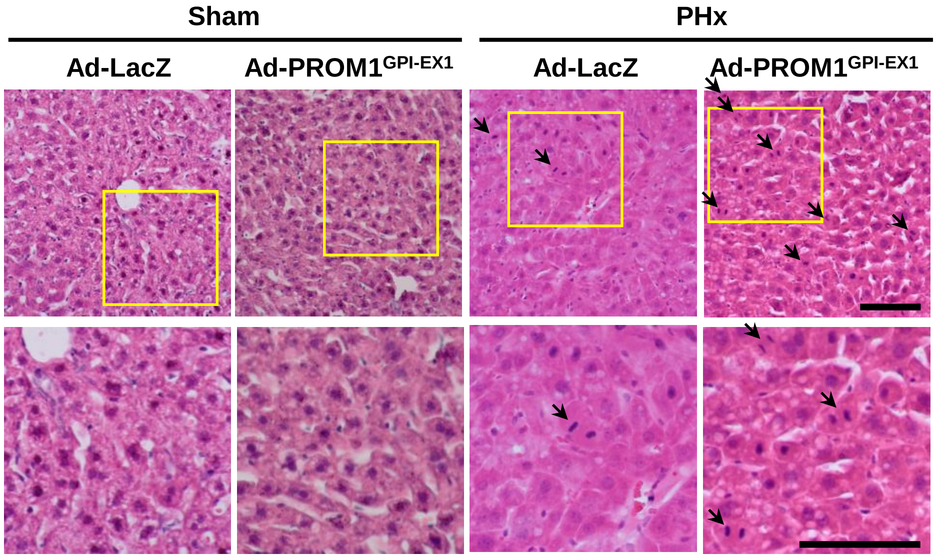

Supplementary Figure 7. Mitotic cells are increased in LacZ and PROM1$^{GPI-EX1}$ -overexpressed *Prom1^{LKO}* livers after partial hepatectomy. A 2/3 partial hepatectomy was performed in 8-week-old male *Prom1^{LKO}* mice after infection with adeno-LacZ or adeno-PROM1$^{GPI-EX1}$-FLAG. Representative H&E staining in the liver 48 hours after PHx. Mitotic cells are indicated by arrows. The lower panels are enlarged images of the yellow boxes in the upper panels. Scale bar = 100 μm.

Name	Forward	Reverse
18S rRNA	CGC CGC TAG AGG TGA AAT TC	CGA ACC TCC GAC TTT CGT TCT
Prom1	GGT GCA CAT CTT CCT CAA CG	CTT GGA ATC AAC TGA GAT GT
Ccnd1	GCG TAC CCT GAC ACC AAT CTC	ACT TGA AGT AAG ATA CGG AGG GC
Ccne1	TCC ACG CAT GCT GAA TTA TC	TTG CAA GAC CCA GAT GAA GA
Ccna2	CTT GGC TGC ACC AAC AGT AA	CAA ACT CAG TTC TCC CAA AAA CA
Ccnb1	GCG TGT GCC TGT GAC AGT TA	CCT AGC GTT TTT GCT TCC CTT
$PROM1^{GPI\text{-}EX1}$	GCA TTC TCT TTG AAC TAG TGC	CTT GTA ATC CCC TGC TTC ATA G
Stat3c	TGA GTC GCT CAC GTT TGA CA	ATC ACC GTC ATG GTC TTT GT
Egf	CGG ACA GCT ACA CGG AAT G	CGA GGC AGA CAC AAA TAA CCC
Hgf	TGT TCC ATG TGG GAC AAG AA	CAA CGG GAA ATA GGG CAA TA

Supplementary Table 1. Primers used in qRT-PCR.

Name	Company (catalog #)	Host	Experiments
PROM1	abcam (ab19898)	Rabbit polyclonal	IP (2ug/1mg lysates)
PROM1	Thermo fisher scientific, eBioscience (14-1331-82)	Rat monoclonal (13A4)	IB (1:500) IF (1:100)
PROM1	Developmental Studies Hybridoma Bank (HB#7)	Mouse monoclonal (HC7)	IB (1:1000)
HNF4α	abcam (ab41898)	Mouse monoclonal (K9218)	IF (1:200)
β-Actin	Santa Cruz Biotechnology (sc-47778)	Mouse monoclonal (C4)	IB (1:1000)
CK19	abcam (ab52625)	Rabbit monoclonal (EP1580Y)	IF (1:200)
Cyclin A	abcam (ab181591)	Rabbit monoclonal (EPR17351)	IB (1:1000)
Cyclin B	Cell Signaling Technology (4138)	Rabbit polyclonal	IB (1:1000)
Cyclin E	Santa Cruz Biotechnology (sc-377100)	Mouse monoclonal (E-4)	IB (1:500)
Cyclin D	Santa Cruz Biotechnology (sc-8396)	Mouse monoclonal (A-12)	IB (1:1000)
PCNA	Cell Signaling Technology (2586)	Mouse monoclonal (PC10)	IB (1:1000)
Ki-67	Cell Signaling Technology (12202)	Rabbit monoclonal (D3B5)	IF (1:200)
P-STAT3	Cell Signaling Technology (9145)	Rabbit monoclonal (D3A7)	IB (1:1000)
STAT3	Cell Signaling Technology (9139)	Mouse monoclonal (124H6)	IB (1:1000)
P-ERK	Cell Signaling Technology (9101)	Rabbit polyclonal	IB (1:1000)
ERK	Cell Signaling Technology (9102)	Rabbit polyclonal	IB (1:1000)
P-AKT	Cell Signaling Technology (9271)	Rabbit polyclonal	IB (1:500)
AKT	Santa Cruz Biotechnology (sc-8312)	Rabbit polyclonal	IB (1:1000)
P-GSK3β	Cell Signaling Technology (9336)	Rabbit polyclonal	IB (1:500)
GSK3β	Cell Signaling Technology (9315)	Rabbit polyclonal	IB (1:1000)
FLAG	Sigma-Aldrich (F7425)	Rabbit polyclonal	IB (1:1000), IF (1:400), IP (2ug/1mg lysates)
FLAG	Sigma-Aldrich (F1804)	Mouse monoclonal (M2)	IB (1:2000)
GP130	Cell Signaling Technology (3732)	Rabbit polyclonal	IB (1:1000)
Flotillin-1	Santa Cruz Biotechnology (sc-25506)	Rabbit polyclonal	IB (1:2000)
Flotillin-1	Santa Cruz Biotechnology (sc-74566)	Mouse monoclonal (c-2)	IB (1:1000)
His	Santa Cruz Biotechnology (sc-53073)	Mouse monoclonal (AD1.1.10)	IB (1:1000), IF (1:200), IP (2ug/1mg lysates)
RFP (tdTom)	Rockland (600-401-379)	Rabbit polyclonal	IF (1:400)
tdTomato	Thermo fisher scientific (TA180009)	Mouse monoclonal (OTI2H2)	IF (1:400)

Supplementary Table 2. Primary antibodies used in immunoprecipitation (IP), immunoblotting (IB) and immunofluorescence (IF) analysis.

Name	Company	Catalog number	Experiments
Goat anti-rabbit IgG (H+L) Secondary antibody, Alexa Fluor 555	Thermo fisher scientific	A21428	IF (1:100)
Goat anti-mouse IgG (H+L) Secondary antibody, Alexa Fluor 488	Thermo fisher scientific	A11001	IF (1:100)
Goat anti-rabbit IgG (H+L) Secondary antibody, Alexa Fluor 488	Thermo fisher scientific	A11034	IF (1:100)
Goat anti-mouse IgG (H+L) Secondary antibody, Alexa Fluor 555	Thermo fisher scientific	A21422	IF (1:100)
Goat anti-mouse secondary antibody, HRP	Santa Cruz Biotechnology	sc516102	IB (1:10000)
Goat anti-rabbit secondary antibody, HRP	Thermo fisher scientific	31460	IB (1:10000)
Goat anti-rat secondary antibody, HRP	abcam	ab97057	IB (1:10000)

Supplementary Table 3. Secondary antibodies used in immunofluorescence (IF) and immunoblotting (IB) analysis.
