# Supplementary material for: Central role of Prominin-1 in lipid rafts during liver regeneration
Source: Nat Commun. 2022 Oct 20;13:6219. doi: 10.1038/s41467-022-33969-4 (PMC9585078; doi:10.1038/s41467-022-33969-4)
Supplement: Supplementary file 3 — Reporting Summary [file 41467_2022_33969_MOESM3_ESM.pdf]

nature portfolio

Corresponding author(s): Young-Gyu Ko

Last updated by author(s): Sep 15, 2022

Reporting Summary

Nature Portfolio wishes to improve the reproducibility of the work that we publish. This form provides structure for consistency and transparency in reporting. For further information on Nature Portfolio policies, see our Editorial Policies and the Editorial Policy Checklist.

Statistics

For all statistical analyses, confirm that the following items are present in the figure legend, table legend, main text, or Methods section.

n/a	Confirmed	
☐	☒	The exact sample size (n) for each experimental group/condition, given as a discrete number and unit of measurement
☐	☒	A statement on whether measurements were taken from distinct samples or whether the same sample was measured repeatedly
☐	☒	The statistical test(s) used AND whether they are one- or two-sided<br>*Only common tests should be described solely by name; describe more complex techniques in the Methods section.*
☒	☐	A description of all covariates tested
☒	☐	A description of any assumptions or corrections, such as tests of normality and adjustment for multiple comparisons
☐	☒	A full description of the statistical parameters including central tendency (e.g. means) or other basic estimates (e.g. regression coefficient) AND variation (e.g. standard deviation) or associated estimates of uncertainty (e.g. confidence intervals)
☐	☒	For null hypothesis testing, the test statistic (e.g. F, t, r) with confidence intervals, effect sizes, degrees of freedom and P value noted<br>*Give P values as exact values whenever suitable.*
☒	☐	For Bayesian analysis, information on the choice of priors and Markov chain Monte Carlo settings
☒	☐	For hierarchical and complex designs, identification of the appropriate level for tests and full reporting of outcomes
☒	☐	Estimates of effect sizes (e.g. Cohen's d, Pearson's r), indicating how they were calculated

Our web collection on statistics for biologists contains articles on many of the points above.

Software and code

Policy information about availability of computer code

Data collection	LightCycler 480 software 1.5.0 (Roche)<br>SoftMaz Pro V6 (Molecular Devices)<br>ZEN 2009
Data analysis	Graphpad Prism 6<br>ImageJ 1.52i<br>ZEN 3.5 blue edition<br>Photoshop (Adobe) CS5

For manuscripts utilizing custom algorithms or software that are central to the research but not yet described in published literature, software must be made available to editors and reviewers. We strongly encourage code deposition in a community repository (e.g. GitHub). See the Nature Portfolio guidelines for submitting code & software for further information.

Data

Policy information about availability of data

All manuscripts must include a data availability statement. This statement should provide the following information, where applicable:

- Accession codes, unique identifiers, or web links for publicly available datasets
- A description of any restrictions on data availability
- For clinical datasets or third party data, please ensure that the statement adheres to our policy

Source data containing uncropped blots and raw data for all plots are provided with this paper.
All other data supporting this study are available within the paper and its supplementary information.

Human research participants

Policy information about studies involving human research participants and Sex and Gender in Research.

Reporting on sex and gender	Not applecable
Population characteristics	Not applicable
Recruitment	Not applicable
Ethics oversight	Not applicable

Note that full information on the approval of the study protocol must also be provided in the manuscript.

Field-specific reporting

Please select the one below that is the best fit for your research. If you are not sure, read the appropriate sections before making your selection.

[x] Life sciences   [ ] Behavioural & social sciences   [ ] Ecological, evolutionary & environmental sciences

For a reference copy of the document with all sections, see nature.com/documents/nr-reporting-summary-flat.pdf

Life sciences study design

All studies must disclose on these points even when the disclosure is negative.

Sample size	The number of mice used in each experiment was determined based on preliminary experiments in the same model.<br>We chose the sample sizes to the smallest statistically significant number to minimize at sacrifice according to animal experiment ethics and Korea University and Korean Animal Protection Law approval.
Data exclusions	No data were excluded from the analysis.
Replication	All experiments were repeated three times independently with similar results.
Randomization	The investigators measured the body weight of mice before the experiments to make sure there is no weight difference between control and experimental groups. To rule out cage-dependent effects, mice were randomly chosen from different cages.
Blinding	The investigators were blinded to group allocation during data collection and analysis.

Reporting for specific materials, systems and methods

We require information from authors about some types of materials, experimental systems and methods used in many studies. Here, indicate whether each material, system or method listed is relevant to your study. If you are not sure if a list item applies to your research, read the appropriate section before selecting a response.

Materials & experimental systems

n/a	Involved in the study
	[x] Antibodies
	[x] Eukaryotic cell lines
[x]	Palaeontology and archaeology
	[x] Animals and other organisms
[x]	Clinical data
[x]	Dual use research of concern

Methods

n/a	Involved in the study
[x]	ChIP-seq
[x]	Flow cytometry
[x]	MRI-based neuroimaging

Antibodies

Antibodies used

PROM1 abcam ab19898 IP 2ug/1mg lysates
PROM1 Thermo fisher scientific 14-1331-82 (13A4) IB 1:500, IF 1:100
PROM1 Developmental Studies Hybridoma Bank HB#7 (HC7) IB 1:1000
HNF4alpha abcam ab41898 (K9218) IF 1:200
beta-Actin Santa Cruz sc-47778 (C4) IB 1:1000
CK19 abcam ab52625 (EP1580Y) IF 1:200
Cyclin A abcam ab181591 (EPR17351) IB 1:1000
Cyclin B Cell Signaling Technology 4138 IB 1:1000
Cyclin E Santa Cruz sc-377100 (E-4) IB 1:500
Cyclin D Santa Cruz sc-8396 (A-12) IB 1:1000
PCNA Cell Signaling Technology 2586 (PC10) IB 1:1000
Ki-67 Cell Signaling Technology 12202 (D3B5) IF 1:200
P-STAT3 Cell Signaling Technology 9145 (D3A7) IB 1:1000
STAT3 Cell Signaling Technology 9139 (124H6) IB 1:1000
P-ERK Cell Signaling Technology 9101 IB 1:1000
ERK Cell Signaling Technology 9102 IB 1:1000
P-AKT Cell Signaling Technology 9271 IB 1:500
AKT Santa Cruz sc-8312 IB 1:1000
P-GSK3beta Cell Signaling Technology 9336 IB 1:500
GSK3beta Cell Signaling Technology 9315 IB 1:1000
FLAG Sigma-Aldrich F7425 IB 1:1000, IF 1:400, IP 2ug/1mg lysates
FLAG Sigma-Aldrich F1804 (M2) IB 1:2000
GP130 Cell Signaling Technology 3732 IB 1:1000
Flotillin-1 Santa Cruz sc-25506 IB 1:2000
Flotillin-1 Santa Cruz sc-74566 (c-2) IB 1:1000
His Santa Cruz sc-53073 (AD1.1.10) IB 1:1000, IF 1:200, IP 2ug/1mg lysates
RFP Rockland 600-401-379 IF 1:400
tdTomato Thermo fisher scientific TA180009 (OTI2H2) IF 1:400
Goat anti-rabbit IgG (H+L) Secondary antibody, Alexa Fluor 555 Thermo A21428 IF 1:100
Goat anti-mouse IgG (H+L) Secondary antibody, Alexa Fluor 488 Thermo A11001 IF 1:100
Goat anti-rabbit IgG (H+L) Secondary antibody, Alexa Fluor 488 Thermo A11034 IF 1:100
Goat anti-mouse IgG (H+L) Secondary antibody, Alexa Fluor 555 Thermo A21422 IF 1:100
Goat anti-mouse secondary antibody, HRP, santa cruz sc516102 IB 1:10000
Goat anti-rabbit secondary antibody, HRP, Thermo 31460 IB 1:10000
Goat anti-rat secondary antibody, HRP, abcam ab97057 IB 1:10000

Validation

PROM1 abcam ab19898 validated in human and mouse for WB, IP and ICC by company
PROM1 Thermo fisher scientific 14-1331-82 validated in mouse for flow cytometry and IF by company
PROM1 Developmental Studies Hybridoma Bank HB#7 validated in Swaminathan et al 2010
HNF4alpha abcam ab41898 validated in human and mouse for IF, IHC and flow cytometry by company
beta-Actin Santa Cruz sc-47778 validated in human and mouse for WB by company
CK19 abcam ab52625 validated in mouse and human for WB, IF, IHC and flow cytometry by company
CyclinA abcam ab181591 validated in mouse and human for WB, IF and IHC by company
Cyclin B Cell Signaling Technology 4138 validated in mouse and human for WB and IF by company
CyclinE Santa Cruz sc-377100 validated in mouse and human for WB and IHC by company
Cyclin D Santa Cruz sc-8396 validated in mouse and human for WB, IF and IHC by company
PCNA Cell Signaling Technology 2586 validated in human and mouse for WB, IHC and flow cytometry by company
Ki-67 Cell Signaling Technology 12202 validated in mouse for IHC by company
P-STAT3 Cell Signaling Technology 9145 validated in human and mouse for WB, IF, IHC and flow cytometry by company
STAT3 Cell Signaling Technology 9139 validated in human and mouse for WB, IF, IHC and flow cytometry by company
P-ERK Cell Signaling Technology 9101 validated in human and mouse for WB, IF and flow cytometry by company
ERK Cell Signaling Technology 9102 validated in human and mouse for WB and IHC by company
P-AKT Cell Signaling Technology 9271 validated in human and mouse for WB, IF and flow cytometry by company
AKT Santa Cruz sc-8312 validated in human and mouse for WB by company

P-GSK3beta Cell Signaling Technology 9336 validated in mouse for WB by company
GSK3beta Cell Signaling Technology 9315 validated in mouse and human for WB and IHC by company
FLAG Sigma-Aldrich F7425 validated in human cells transfected with FLAG tagged fusion proteins for WB and IF company
FLAG Sigma-Aldrich F1804 validated in canine cells transfected with FLAG tagged myr-PHCz for IF by company
GP130 Cell Signaling Technology 3732 validated in human and mouse for WB by company
Flotillin-1 Santa Cruz sc-25506 validated in human and mouse for WB and IF by company
Flotillin-1 Santa Cruz sc-74566 validated in human and mouse for WB and IHC by company
His Santa Cruz sc-53073 validated in human cells transfected with His-tagged fusion proteins for WB by company
RFP Rockland 600-401-379 validated in human and mouse cells and tissues transfected with RFP tagged fusion proteins for WB, IF and IHC by company
tdTomato Thermo fisher scientific TA180009 validated in human cells transfected with tdTomato tagged fusion proteins for WB by company
Goat anti-rabbit IgG (H+L) Secondary antibody, Alexa Fluor 555 Thermo A21428 validated for IF by company
Goat anti-mouse IgG (H+L) Secondary antibody, Alexa Fluor 488 Thermo A11001 validated for IF by company
Goat anti-rabbit IgG (H+L) Secondary antibody, Alexa Fluor 488 Thermo A11034 validated for IF by company
Goat anti-mouse IgG (H+L) Secondary antibody, Alexa Fluor 555 Thermo A21422 validated for IF by company
Goat anti-mouse secondary antibody, HRP, santa cruz sc516102 validated for WB by company
Goat anti-rabbit secondary antibody, HRP, Thermo 31460 validated for WB by company
Goat anti-rat secondary antibody, HRP, abcam ab97057 validated for WB by company

Eukaryotic cell lines

Policy information about cell lines and Sex and Gender in Research

Cell line source(s)	Primary mouse hepatocytes were isolated from 8-week-old male C57BL/6 mice.<br>HEK 293 cells (ATCC CRL-3216)<br>AD293 cells (ATCC)
Authentication	Primary mouse hepatocytes were authenticated by analysis of specific cell marker (HNF4alpha).<br>HEK 293 cells and AD293 cells were authenticated using Short Tandem Repeat (STR) profiling by ATCC.
Mycoplasma contamination	Primary mouse hepatocytes were not tested for mycoplasma.<br>HEK 293 cells and AD293 cells were negative of mycoplasma contamination.
Commonly misidentified lines<br>(See ICLAC register)	No misidentified cell lines were used in the study.

Animals and other research organisms

Policy information about studies involving animals; ARRIVE guidelines recommended for reporting animal research, and Sex and Gender in Research

Laboratory animals	8-week-old C57BL/6 male mouse (Mus musculus)
Wild animals	Not involved wild animals.
Reporting on sex	The study did not conduct sex-based analysis. Previous report (Baatarsuren Batmunkh et al., 2017. Acta Histochem Cytochem. doi: 10.1267/ahc.17003) showed that estrogen promotes liver regeneration. Since we considered only the liver regenerative function of PROM1, excluding the hormonal effects, only male mice were used in the experiments.
Field-collected samples	The study did not involve samples collected from the field.
Ethics oversight	Approved by the Institutional Animal Care and Use Committee of Korea University and the Korean Animal Protection Law (KUIACUC-2019-0111)

Note that full information on the approval of the study protocol must also be provided in the manuscript.
